# Current Understanding of Residual Force Enhancement: Cross-Bridge Component and Non-Cross-Bridge Component

**DOI:** 10.3390/ijms20215479

**Published:** 2019-11-04

**Authors:** Atsuki Fukutani, Walter Herzog

**Affiliations:** 1 Faculty of Sport and Health Science, Ritsumeikan University, 1-1-1 Noji-higashi, Kusatsu, Shiga 525-8577, Japan; 2 Faculty of Kinesiology, The University of Calgary, 2500 University Drive, NW, Calgary, AB T2N 1N4, Canada; wherzog@ucalgary.ca

**Keywords:** actin, myosin, titin, sarcomere length non-uniformity, force-length relationship

## Abstract

Muscle contraction is initiated by the interaction between actin and myosin filaments. The sliding of actin filaments relative to myosin filaments is produced by cross-bridge cycling, which is governed by the theoretical framework of the cross-bridge theory. The cross-bridge theory explains well a number of mechanical responses, such as isometric and concentric contractions. However, some experimental observations cannot be explained with the cross-bridge theory; for example, the increased isometric force after eccentric contractions. The steady-state, isometric force after an eccentric contraction is greater than that attained in a purely isometric contraction at the same muscle length and same activation level. This well-acknowledged and universally observed property is referred to as residual force enhancement (rFE). Since rFE cannot be explained by the cross-bridge theory, alternative mechanisms for explaining this force response have been proposed. In this review, we introduce the basic concepts of sarcomere length non-uniformity and titin elasticity, which are the primary candidates that have been used for explaining rFE, and discuss unresolved problems regarding these mechanisms, and how to proceed with future experiments in this exciting area of research.

## 1. Introduction

Muscle contraction is essential for producing movements. Therefore, the mechanism underlying muscle contraction has been extensively studied in an effort to understand the physical behavior of living organisms. Currently, the cross-bridge theory, introduced by A.F. Huxley [1] has been widely accepted as the reigning paradigm for the molecular mechanisms producing muscle contraction. Specifically, the sliding of actin filaments relative to myosin filaments, which produces muscle shortening and force, is produced by the cyclic attachment of myosin-based cross-bridges (originally referred to as “side-pieces”, Huxley [1]) to specific sites on the actin filaments. Later, Rayment et al. [2] revealed that rotation of the myosin head, which has been identified as the “power stroke,” generates the sliding of actin filaments relative to myosin filaments coupled with the hydrolysis of ATP. Thus, the myosin heads attached to the actin filament produce muscle force. Muscle forces estimated by the cross-bridge theory agree well with experimentally observed muscle properties, such as the concentric part of the force-velocity relationship [3] and the force-length relationship [4], leading to the acceptance of the cross-bridge theory as the paradigm for the molecular events underlying skeletal muscle contraction.

**Figure 1:**
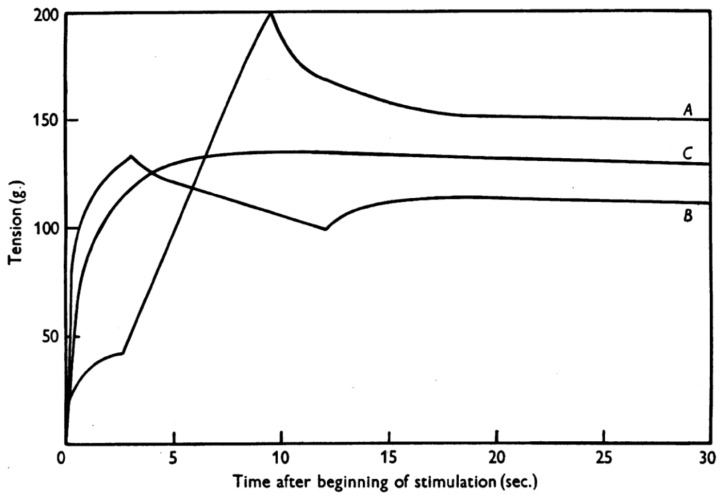
Residual force enhancement and residual force depression [5]. Force was measured in dogfish muscles. Note that the isometric steady-state force after stretch (**A**) and after shortening (**B**) were different compared to the force measured in a purely isometric contraction (**C**) although the final muscle length and contraction (stimulation) intensity were identical among A, B and C. (Reprinted with permission).

However, many experimental observations and basic muscle properties cannot be explained well with the cross-bridge theory. For example, the eccentric part of the force-velocity relationship and the history-dependent properties are not well captured, or cannot be explained at all with the cross-bridge theory. Specifically, the increase in isometric force after eccentric contractions remains unexplained. Steady-state, isometric forces after an eccentric contraction are greater than the isometric, steady-state forces of purely isometric contraction at the corresponding muscle length and activation level [5]. According to the cross-bridge theory, muscle forces at steady-state are predicted to be identical at a given muscle length and activation level because the number of attached cross-bridges, and their average force is predicted to be identical, independent of the history of contraction [1,4]. This phenomenon is referred to as residual force enhancement (rFE) [5,6,7] (Figure 1), and it has been observed consistently and at all structural levels of muscle [5,8,9,10,11,12,13,14,15,16,17]. However, the molecular mechanisms underlying rFE remain largely unknown, although experimental observations and evidence have accumulated rapidly in the past few years, resulting in a myriad of proposed mechanisms that await stringent evaluation. In the following, we first introduce the basic concept of the cross-bridge theory, and then introduce why rFE cannot be explained by the cross-bridge theory. Next, we introduce the two primary mechanisms that have been used to explain rFE: (i) the sarcomere length non-uniformity theory, and (ii) the titin theory. The basic concepts, experimental support, and remaining problems of these mechanisms are discussed in some detail with the hope to provide a better understanding of the molecular mechanisms underlying muscle contraction.

## 2. Cross-Bridge Theory

**Figure 2:**
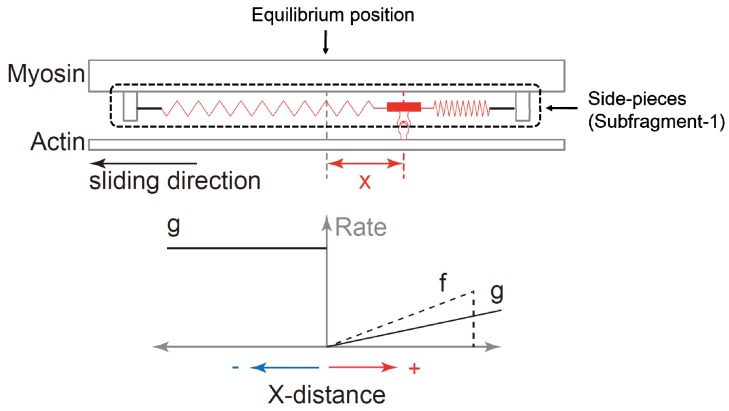
Schematic diagram of the cross-bridge theory proposed by A.F. Huxley [1]. The upper panel shows the structural model of the actin and myosin, and the lower panel shows the functional model of attachment and detachment rates suggested by A.F. Huxley [1]. When the myosin subfragment-1 (side-pieces) attaches to the actin filament, the actin filament is moved past myosin because of the elastic force produced by the springs that attach the cross-bridges to the backbone of the myosin filament. This elastic force (the force produced by the cross-bridge) depends on the distance of the nearest actin attachment site (indicated by the knob on the actin filament) to the equilibrium position (x-distance) of the cross-bridge. The rate constant of attachment and detachment is also dependent on the x-distance (the left lower panel). The vertical axis means the rate (probability) of attachment or detachment. Finally, the force of an entire muscle produced by the cross-bridges depends on the average force of the attached cross bridges and the number of attached cross-bridges.

H.E. Huxley and Hanson [18] and A.F. Huxley and Niedergerke [19] reported in the same issue of *Nature* that the length of the sarcomere A-band remained virtually constant for a variety of contractile conditions, while the length of the I-band changed substantially. Based on these findings, they suggested that muscle contraction occurred through the relative sliding between actin and myosin filaments, rather than the shortening of the myosin filament, as assumed at that time. A.F. Huxley [1] then proposed a molecular framework that could produce this relative sliding between the two sets of filaments (Figure 2). He proposed that the myosin filament has side-pieces (now identified as the myosin subfragment-1) that are connected to the myosin filament backbone through an elastic spring. These side-pieces were proposed to attach to and detach from the actin filament with defined rate constants that depended exclusively on the distance of the cross-bridge equilibrium position to the nearest eligible actin attachment site (Huxley’s so-called x-distance). When the x-distance increases, the force produced by each cross-bridge (which depends on the extension of the cross-bridge spring) becomes greater too. In addition, when the number of attached cross-bridges increases, the force produced by the muscle becomes greater too. Gordon et al. [4] confirmed that the number of possible cross-bridge attachments was crucial for force production. They measured the isometric force at various sarcomere lengths, and predicted that the active sarcomere force decreases linearly with the loss of actin-myosin filament overlap on the descending limb of the force-length relationship, because of a corresponding decrease in the potential cross-bridge interactions between actin and myosin. Their prediction was supported by experiment indicating that the proposed molecular mechanism by A.F. Huxley [1] is feasible. In addition, the rate constants in the cross-bridge theory were adjusted in such a manner that the force-velocity properties of skeletal muscles [3] could be predicted too.

## 3. Residual Force Enhancement (rFE)

The cross-bridge theory can nicely explain the mechanical force responses for isometric and concentric contractions [1]. However, some basic and consistent experimental observations cannot be explained by the cross-bridge theory. For example, according to the cross-bridge theory, the steady-state, isometric force at a given sarcomere length should always be the same, because the number of attached cross-bridges and the average force per cross-bridge are predicted to be the same. However, Abbott and Aubert [5] found that the steady-state, isometric force at a given muscle length differed depending on the history of muscle contraction. Specifically, once muscles were elongated (or shortened) actively, the steady-state, isometric force after the elongation (or shortening) was greater (or smaller) than the corresponding purely isometric reference force attained at the same muscle length and same activation level (Figure 1). This fundamental property of skeletal muscle has been labelled rFE and residual force depression. These history-dependent force responses cannot be predicted by the classic cross-bridge theory [1,4], indicating that the currently accepted molecular mechanism of muscle contraction, the cross-bridge theory, is either incorrect or incomplete.

Following Abbott and Aubert’s classic work [5], rFE has been extensively studied across multiple structural scales, including single sarcomeres [14,15], single myofibrils [10,12], single intact fibers [6,7], single skinned fibers [16,17], isolated whole muscles [5,13], and human joints [9,11]. Since single sarcomere preparations were shown to produce vast rFE, this property must be considered a sarcomeric property that is, at least in part, related to the contractile and/or structural proteins in sarcomeres. Based on abundant experimental evidence, the following characteristics have been identified; rFE is prominent on the descending limb of the force–length relationship [20,21,22], increases with increasing stretch magnitudes [6,23,24], and is largely independent of the stretch speed [8,25]. However, although many studies on rFE have been conducted, the molecular mechanism(s) underlying rFE remain to be clarified. Several mechanisms have been proposed. Here, we introduce the two primary mechanisms: (i) the sarcomere length non-uniformity and (ii) the titin mechanism, and then, proceed with a critical discussion of the two.

## 4. Sarcomere Length Non-Uniformity

### 4.1. Concept

**Figure 3:**
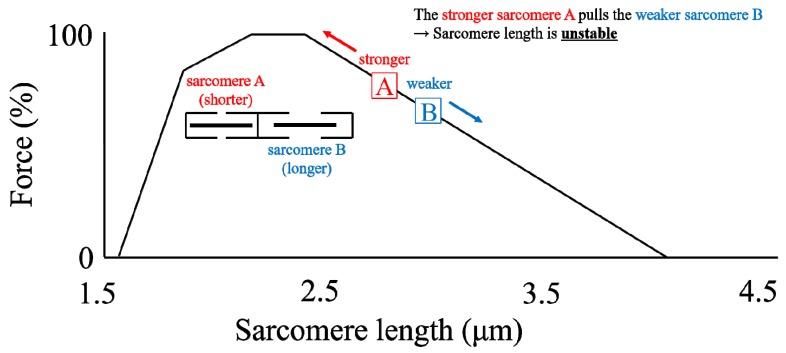
Schematic diagram of two non-uniform sarcomeres on the descending limb of the force-length relationship. Note that the shorter sarcomere A has a larger force-generating capacity than the longer sarcomere B. Thus, the shorter sarcomere A is thought to stretch the longer sarcomere B upon muscle contraction.

**Figure 4:**
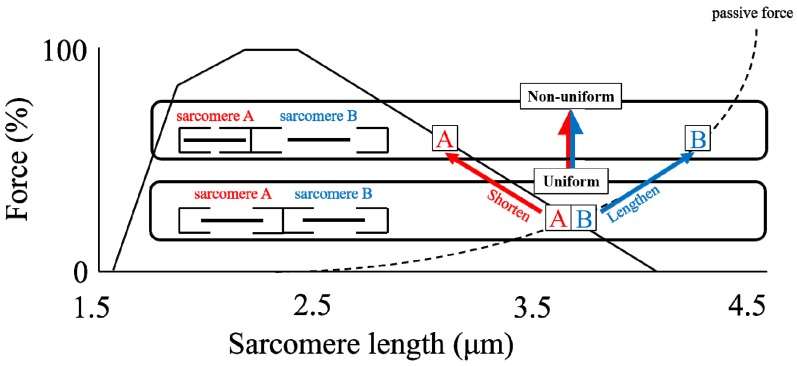
Schematic diagram of two (1) uniform (in length) sarcomeres (lower) and (2) non-uniform (in length) sarcomeres (upper) on the descending limb of the force-length relationship. These two sarcomeres are stabilized because they have the same force-generating capability for both conditions. Note that even though the sum of the length of sarcomeres A and B is identical between the two conditions, the force produced by these conditions is different.

By far the most accepted and longest lasting proposal for explaining rFE is the sarcomere length non-uniformity theory [20,26]. This mechanism is based on the idea that sarcomere lengths and forces are unstable on the descending limb of the force-length relationship, as first suggested by Hill [27], and then reinforced by others [4,21,28,29]. The argument goes as follows: Imagine two identical sarcomeres on the descending limb of the force-length relationship at slightly different lengths (Figure 3). The shorter sarcomere has greater actin-myosin filament overlap and thus will tend to shorten, while the longer sarcomere has less actin-myosin filament overlap than the short sarcomere, and thus is thought to be stretched by the short sarcomeres. This imbalance continues until the stretched sarcomere becomes so long that passive forces will help it reach a force equilibrium with the short sarcomere. The non-uniformity in sarcomere length then leads to a situation where the equilibrium force of the two non-uniform sarcomeres is greater than the theoretical force at the average sarcomere length for these two sarcomeres (Figure 4). This difference in equilibrium force between the non-uniform sarcomeres (Figure 4, upper) and the theoretical uniform sarcomeres (Figure 4, lower) is then thought to produce the enhanced force following an active stretch, because it is assumed that active muscle stretching will produce these sarcomere length non-uniformities while mere isometric contractions will not.

### 4.2. Problems

If sarcomere length non-uniformity is assumed to be the exclusive factor for explaining rFE, then several testable assumptions can be derived. First, rFE should only be observed on the (unstable) descending but not the (stable) ascending limb of the force-length relationship. Second, the isometric force in the enhanced state (rFE state) should not exceed the maximal isometric reference force obtained at optimal muscle/sarcomere length. Third, rFE should not occur in a single sarcomere preparation. Fourth, sarcomere lengths should be highly non-uniform in the rFE state and essentially uniform in the isometric reference state.

Regarding the first point, rFE should only be observed on the (unstable) descending, but not the (stable) ascending limb of the force-length relationship [20,26,27]. However, rFE has been observed on the ascending limb ever since the classic study by Abbott and Aubert [5], although it is acknowledged that rFE is typically smaller on the ascending than the descending limb [21,22,30]. These results indicate that at least some parts of the rFE cannot be explained by sarcomere length non-uniformity. Regarding the second point, the theoretical limit of rFE in the sarcomere length non-uniformity theory is given by the maximal, steady-state, isometric force of a muscle in the non-enhanced, purely isometric reference state. This is because some of the force in the rFE state must come from sarcomeres that produce active force and are not over-stretched [7,21,31]. However, experimental results indicate that forces in the enhanced state exceeded this theoretical limit [14,22,30,31,32], thereby violating a basic prediction of the sarcomere length non-uniformity theory. Regarding the third point, at least two sarcomeres are needed to induce sarcomere length non-uniformity, thus a single sarcomere cannot produce rFE. However, Leonard et al. [14], and Rassier and Pavlov [15] showed that a mechanically isolated single sarcomere can produce large amounts of rFE and forces above the isometric plateau of the force-length relationship [14]. These results indicate that rFE is, at least in part, related to contractile and/or structural proteins in the sarcomere, and can be produced in the absence of sarcomere length non-uniformities. Sometimes, the argument has been made that half-sarcomere length non-uniformity in a single sarcomere may produce the observed rFE. However, such an argument fails logically, as a single sarcomere has only 1 degree of freedom for half-sarcomere lengths. Therefore, one of the two halves of a sarcomere must be producing force in the actin-myosin filament overlap zone (while the other half sarcomere may be over-stretched), and the half sarcomere in the overlap zone must produce a force greater than the isometric force, and greater than the isometric plateau force, which proves that the rFE property must also be in a half-sarcomere.

**Figure 5:**
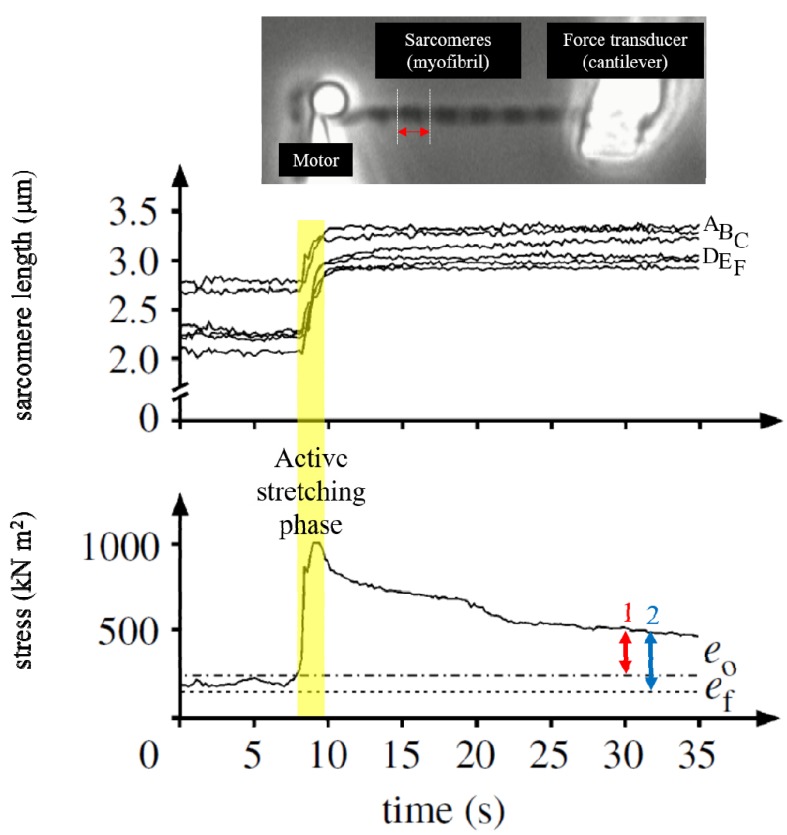
Sarcomere lengths changes before and after active stretching in a single myofibril preparation [12]. This myofibril had six sarcomeres and each sarcomere length was monitored throughout the trial. Thus, there are six traces in the upper graph. The steady-state isometric force after stretch was larger than the purely isometric force obtained at the same sarcomere length (*e*_f_, 2 and the force measured at the optimal sarcomere length (*e*_0_, 1) (lower panel). The increased isometric force, indicated by the blue arrow, represents the magnitude of rFE. If rFE was caused by the development of sarcomere length non-uniformities, this enhanced isometric force should not exceed the isometric force attained at the optimal length. However, the isometric force in the enhanced state was greater than the isometric force at optimal sarcomere length, as indicated by the red arrow). In the enhanced force state, sarcomere length was indeed different among sarcomeres, but this difference seems to be stable, and similar sarcomere length differences were also observed in the purely isometric contraction (non-enhanced state). (Reprinted with permission).

Together, the overwhelming experimental evidence suggests that rFE can be obtained in the absence of sarcomere length non-uniformity. However, this leaves the possibility that sarcomere length non-uniformity may partly contribute and enhance rFE. A crucial assumption in the sarcomere length non-uniformity theory is that sarcomere length becomes non-uniform by active muscle stretching and sarcomere length is uniform in purely isometric contractions. Although there is good evidence that sarcomere lengths are non-uniform in a muscle after active stretching, there is also overwhelming evidence that such non-uniformities also exist in purely isometric contractions [33,34,35]. Joumaa et al. [12] revealed that the sarcomere lengths in the rFE state of myofibrils were indeed non-uniform, but they were perfectly stable (Figure 5). Similarly, Johnston et al. [36] found that sarcomere length non-uniformities, albeit present in myofibril preparations in the rFE state, were not greater than those observed in the isometric reference contractions. In other words, sarcomere length non-uniformity was about the same for isometric and rFE state contractions. Furthermore, sarcomere lengths stability (assessed by sarcomere dispersion over time) in single myofibrils and fibers were significantly better in the rFE state than those measured for purely isometric reference contractions [7,12,36]. Although non-homogeneous length changes have been reported in intact single fibers [7,25], these measures were “segment lengths” rather than “sarcomere lengths.” Since the myofibril preparations that were used for confirming stable sarcomere length behavior in the rFE state included only about ten serial sarcomeres, and since the influence of inter-sarcomere coordination may be expected to be more prominent when the number of sarcomeres in series is large, it may be possible that a large-scale preparation that contains hundreds of sarcomeres in series shows inhomogeneous sarcomere length changes. Because of the recent advances in technology, individual sarcomere lengths can be measured in whole muscle preparation [34,35] and in intact human skeletal muscles [33,37,38]. However, they have not been used to compare sarcomere length changes and sarcomere length stability in isometric and rFE muscle states.

## 5. Titin Elasticity

### 5.1. Concept

**Figure 6:**
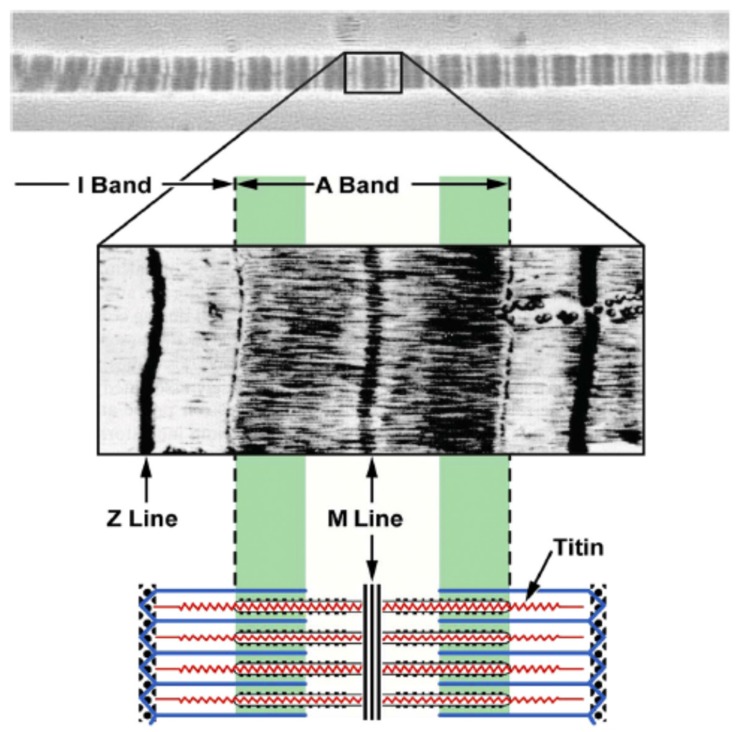
The location of titin in the sarcomere [46]. Titin spans from the Z line to M line. Titin in the A band region is strongly connected with the myosin filament so that this region cannot be elongated during stretch. In contrast, titin in the I band region acts like a molecular spring and accommodates elongations of sarcomeres (Reprinted with permission).

As discussed above, many of the most basic predictions of the sarcomere length non-uniformity theory have been shown to be violated by experimental observations. Furthermore, it has been shown convincingly that rFE can occur in the absence of sarcomere length non-uniformities. Therefore, there must exist another mechanism that either fully or partly explains the origins of rFE. Based on the existence of rFE in single sarcomere and half-sarcomere preparations [14,15], it is likely that sarcomeric proteins contribute to rFE. A frequently-mentioned mechanism for explaining rFE is the engagement of a passive elastic structure upon muscle activation. This is an appealing mechanism because it could explain that rFE increases with increasing stretch magnitude but is essentially independent of the speed of muscle stretching [7]. Early on in this area of research, titin was proposed as being perfectly placed to take on the role of this passive structural element that is engaged upon muscle activation and stretch and can produce rFE [39]. Theoretical models using titin as the structure that causes force enhancement in one way or another have been abundant ever since [40,41,42,43]. Titin (or connectin) is a structural protein in the sarcomere that spans from the Z line to the M band [44,45,46] (Figure 6). Because of its location in the sarcomere, titin produces elastic force when the sarcomere is elongated. Interestingly, this elastic force can be modulated by various processes [47]. Candidates for the modulation of titin stiffness, among many others, are calcium [48,49,50], phosphorylation [51,52], and increased effective titin stiffness because of the shortening of titin’s free spring length [49,53,54]. The total force produced by a sarcomere is the sum of the active cross-bridge-induced forces and the forces produced by parallel elastic muscle components. If the forces produced by parallel elastic components increase upon activation and active muscle lengthening, rFE may be achieved without changing the cross-bridge-induced force [47,53,54,55,56]. The proposal of titin as an activatable spring has many advantages and essentially could explain all hitherto made observations on rFE properties of skeletal muscles. Specifically, this mechanism would allow for rFE on the ascending limb of the force-length relationship, and rFE would be predicted to be small at these sarcomere lengths because titin would not be strained or only strained to a small degree. Also, titin-based rFE could readily explain forces in the enhanced state that exceed the maximal isometric force at optimal muscle length, because titin force would contribute in addition to the cross-bridge-based forces. Furthermore, the observed rFE in single sarcomeres could be explained by titin because titin is a structural protein inside sarcomeres. Therefore, it appears that titin “engagement” upon muscle activation and stretch, could be a feasible mechanism to explain the rFE property of skeletal muscle.

### 5.2. Problems

**Figure 7:**
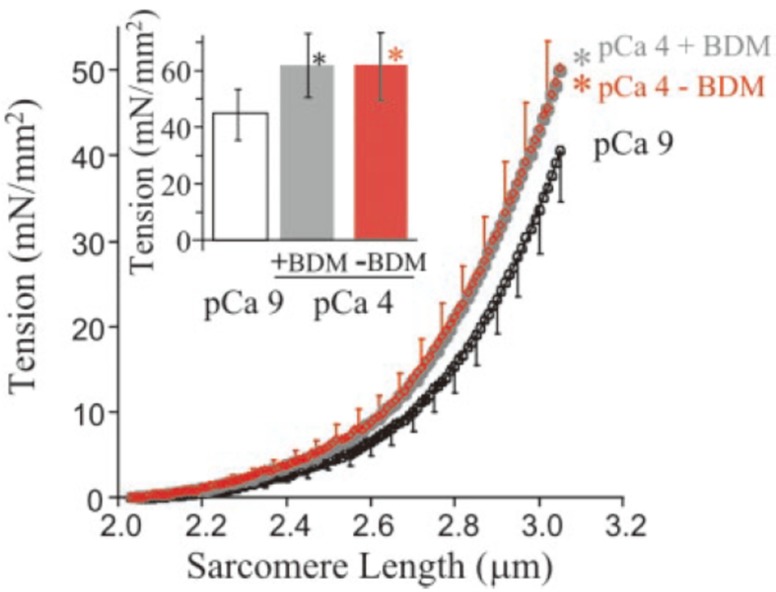
Increased passive force by adding Ca^2+^ [48]. The passive force in the skinned fibers (mainly derived from titin) was increased by adding Ca^2+^ (pCa4.0). This enhancement was not different although the cross-bridge cycling was inhibited by adding 2,3-butanedione monoxime (BDM), indicating that this enhanced force was caused by Ca^2+^. (Copyright (2003) National Academy of Sciences).

**Figure 8:**
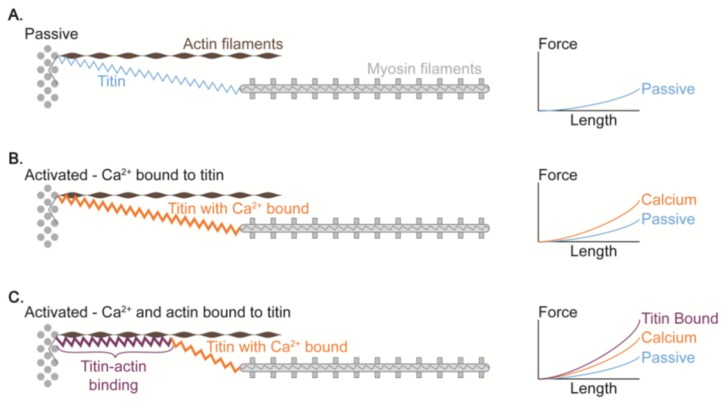
Proposed mechanism of titin-actin interaction [63]. Compared to the normal condition A (i.e., no Ca^2+^ and no titin-actin binding), the passive force is greater in the Ca^2+^-induced stiff titin condition B. This passive force is further increased when the titin is attached to the actin because the titin stiffness and force at a given sarcomere length should increase (condition C).

As introduced above, the elastic force produced by titin can be modulated. If this modulation indeed was shown to occur during active muscle lengthening, then this concept could explain the rFE property without the need for changing the basic assumptions of the cross-bridge theory. At present, several mechanisms have been proposed for explaining the modulation of elastic force of titin. First, Ca^2+^ binding to the titin is a possible mechanism. Labeit et al. [48] showed in skinned fiber preparations that adding Ca^2+^ increased the titin-based force. They confirmed that the magnitude of the increase in titin elastic force was the same for conditions when fibers were exposed or not exposed to 2,3-butanedione monoxime, an agent that has been shown to inhibit strongly bound cross-bridge attachments [57] (Figure 7). This result indicates that Ca^2+^ release itself can contribute to the observed increased elastic force in titin. Similarly, Joumaa et al. [12] reported that the force in titin was increased after active lengthening of single myofibrils in which the regulatory protein troponin C was chemically removed to prevent cross-bridge cycling upon activation. However, the increase in titin force was substantially smaller compared to the same stretch conditions with troponin C intact [58]. These results suggest that titin force increases upon muscle activation (i.e., an increase in [Ca^2+^]), but that the increases are relatively modest compared to the situation in which normal cross-bridge action is allowed. In order to explain the vastly increased titin force upon active muscle stretching in the presence of normal cross-bridge cycling, another mechanism for rFE has been proposed: a shortening of titin’s free spring length by titin (proximal segment) binding to actin [59] (Figure 8). Titin-actin binding seems a feasible proposal, as it has been reported that the actin filament has several potential binding sites for titin [60,61,62]. Once (the proximal segment of) titin is bound to actin, titin stiffness and force at a given sarcomere length would increase. Specifically, it has been proposed that titin’s so-called PEVK (proline, glutamate, valine and lysine) region attaches to the actin filament [61,62]. If so, the proximal Ig domains would be prevented from elongating during active stretch. Instead, only the Ig domains distal to the PEVK region would be available for elongation of sarcomeres, thereby increasing the strain in these elements and providing increased force in the titin filaments [53,63,64]. If this titin–actin binding is induced by eccentric contraction-specific events, this idea well explains the observed mechanical responses regarding rFE. However, observations contradicting this idea have been reported. For example, Cornachione et al. [65] observed rFE for conditions where actin filaments were extracted by gelsolin, indicating that titin-actin interaction is not needed for inducing rFE, supporting the results by Labeit et al. [48] and Joumaa et al. [12] who showed rFE (albeit a small amount) in the absence of actin-myosin interactions, likely caused by a stiffening of titin through Ca^2+^ binding to titin’s E-rich region in the PEVK segment [48] and Ca^2+^ binding to selected immunoglobulin domains [50]. Therefore, rFE seems to have at least two components: (i) a component with a small, sometimes even negligible effect associated with Ca^2+^ activation, tentatively thought to be caused by Ca^2+^ binding to specific sites on titin, and (ii) a component of unknown origin that has a much greater effect, and is tentatively associated with titin binding to actin upon muscle activation and stretch.

Some results on rFE in striated muscles remain controversial: for example, the question if cardiac muscle has rFE properties. Cardiac titin isoforms are smaller than skeletal muscle titin [65,66,67], and as such are also much stiffer and carry a much greater force, at a given sarcomere length, than skeletal muscle titin [65,66,67]. The different structure and function of cardiac titin compared to skeletal muscle titin might also change some of its properties, for example, the ability to contribute to rFE. Cornachione et al. [65], reported that cardiac myofibrils did not have the capacity for rFE, but their raw data traces show great passive force enhancement in cardiac myofibrils. Since passive force enhancement is always associated with rFE, there is a logical contradiction in their results [23,32,55]. Also, their stretch speed for the cardiac myofibrils was extremely fast, causing what appeared to be slippage in the raw data records, which if correct, would have prevented rFE from occurring [68]. In the meantime, unpublished results from our own lab unequivocally suggest that cardiac muscle has rFE properties similar to skeletal muscles. Taken this situation into account, the results by Cornachione et al. [65] should be considered with great caution, and the inconsistency in their result figures and discussion needs to be kept in mind when interpreting their results [69,70,71,72]. So far, systematic and well-executed results from cardiac myofibrils are missing to resolve this controversy, requiring future research.

One of the keys to resolve the detailed contribution of titin to rFE would be to precisely determine the deformation of titin during and after active stretch. Moreover, other factors, such as the phosphorylation and oxidation of titin molecules [73,74] are also known to change titin stiffness. Thus, the influence of these “activation-dependent” chemicals should be examined systematically. However, what we should be aware of is that these chemicals are released not only in eccentric contraction but also in isometric and concentric contractions. Thus, it is difficult to consider that these activation-dependent chemicals alone are the key factor for the increased titin stiffness, i.e., rFE, although they may play an important role in isometric, concentric, and eccentric contractions other than rFE. We speculate that the effect of the activation-dependent chemicals on titin (rFE) may work in connection with the cross-bridge kinetics specific to eccentric contractions. Therefore, the cross-bridge behavior during eccentric contractions, and associated chemical reactions, should be evaluated simultaneously in the future to potentially gain crucial insights into the mechanisms of rFE.

Another question that should be answered systematically is if stiffness of sarcomeres increases in the force-enhanced states compared to the corresponding isometric reference state. If increased titin stiffness is indeed the cause for rFE, one might expect that sarcomere stiffness should also be enhanced in the force enhanced compared to the isometric reference states. Sugi and Tsuchiya [25] determined the stiffness of single fiber preparations in the enhanced and non-enhanced states. They found that stiffness was decreased in the force enhanced compared to the isometric reference states. They further confirmed by using the X-ray diffraction that the 1.1 intensity decreased while the 1.0 intensity was not changed, and interpreted that this decreased intensity ratio of 1.1/1.0 may be caused by a disordering of the myosin filaments leading to an increased repulsion force [75]. However, others found no increase in muscle/fiber stiffness [20], and some report an increase in stiffness in the force enhanced compared to the isometric reference states [76], thus providing no clear answer. If the observation by Sugi and Tsuchiya [25] were indeed correct, an increased titin stiffness/force alone may not explain rFE. However, since cross-bridge stiffness (about 1 pN/nm, Percario et al. [77]) is about 100 times greater than titin stiffness (0.01 pN/nm, Kellermayer et al. [78]), even a doubling or tripling of titin stiffness would barely be measurable in a muscle stiffness test. Since Sugi and Tsuchiya [25] measured fiber stiffness close to the optimal length, stiffness would be dominated by the attached cross-bridges, and not titin. To further examine this, it might be useful to measure the muscle/fiber/sarcomere stiffness at sarcomere lengths where cross-bridge forces are minimal, for example, longer than 3.0 μm in single frog or longer than 3.5 µm for rabbit skeletal muscles. For these sarcomere lengths, the contribution of cross bridge stiffness to the total muscle stiffness may be sufficiently low for changes in titin stiffness to be measurable. Another way to explore the stiffness contributions of titin and cross-bridges may be using low angle X-ray diffraction. When the number of attached cross bridges increases, the intensity ratio of 1.1/1.0 confirmed on the equatorial X-ray reflections increases [79], which is not affected by titin stiffness. Thus, if the muscle stiffness increases without a change in the intensity ratio of 1.1/1.0, this result would lend support that titin stiffness may contribute to the increased muscle stiffness.

## 6. Conclusions

Although the cross-bridge theory nicely explains the mechanical properties of isometric and concentric contractions, it does not explain well the mechanics of eccentric contractions and associated rFE. Based on the current evidence, titin seems to be a promising candidate for explaining the mechanisms underlying rFE, while sarcomere length instability and non-uniformity are likely not involved in contributing to rFE. Although the detailed molecular events causing rFE in skeletal muscle are not resolved, it is likely that titin, together with cross-bridge action that includes the strongly bound cross-bridge state, are the major contributors to rFE. Titin is a spring that can adapt its stiffness in an activation and cross-bridge force-dependent manner in such a way that it can explain rFE beautifully without changing the basic pillars/assumptions of the cross-bridge theory. rFE is a basic property of skeletal and passively cardiac muscles. Recent evidence suggests that in contrast to the long-held believes, rFE is not associated with the development of instability and sarcomere length non-uniformity, but rather, is a property of the contractile machinery of a sarcomere. From a fundamental point of view, this changes how we might think about muscle contraction in the future, and the traditional two filament model of muscle contraction (actin and myosin) will be replaced by a three filament sarcomere that also includes titin as a force regulatory and “contractile” protein.

rFE has been shown to be associated with a substantial reduction in the metabolic cost of force production [16], and as such, the primary evolutionary reason for rFE may not be the increased force one can obtain following active muscle stretching, but the reduction in metabolic cost. This would be of great advantage for muscles in performing work with less energy than otherwise possible if titin and rFE did not exist. rFE also plays a role in everyday sports activities. When we throw an instrument, we do that with a counter-movement that often starts with an active eccentric lengthening of the muscle, thereby increasing the force a muscle could otherwise produce, and then this increased force (and increased work capacity) of the muscle can be used to enhance athletic movements and athletic performance. In addition, it has been shown that elderly people maintain eccentric muscle force better than concentric or isometric force [80,81]. It is likely that this is possible because in concentric and isometric contraction, titin’s role is likely negligible, while in eccentric contraction it is likely not. Therefore, titin, and its function in actively lengthening muscle may be a mechanism to reduce frailty and function in the elderly. For cardiac muscle, there appears to be an active elongation of the sarcomeres just toward the end of diastole, and if so, this could then activate rFE and allow the heart to beat with more force, increase its working capacity, and do so at reduced energetic cost.
